# Annotations

**Published:** 1905-01-28

**Authors:** 


### ANNOTATIONS.

### The Mansion House Committee on the Unemployed.

PROBABLY there is no class of the community better fitted to appreciate the ill effects of an undisciplined charity than those medical practitioners whose lot it is to practise among the poorer classes in our large towns. Their work carries them behind the scenes, and remote not less than immediate results come under their observation. The stories coming from many districts during recent weeks have been sad enough in all conscience, and must mean for many an overworked practitioner a state of affairs, which may well command the sympathy of those more fortunately situated. But it is impossible to doubt that some well-meant efforts to relieve distress carry with them unfortunate consequences. Enthusiastic philanthropists often fail to understand that efficient help means something more than food and fuel, and that the supply of these under the promptings of compassion may destroy the self-respect necessary for the maintenance of true manhood. Those who work amongst and understand the labouring poor could tell many tales of the ill results which have followed an exploitation of their sufferings by competitive philanthropists. It is so far satisfactory to know that facts of this order have been appreciated, at least to some extent, by those concerned with the problem of the unemployed, and that the Central Committee has within a few weeks of the date of its creation, been able, either directly or through the borough committees, to put to work over 2,000 applicants, and to found an organisation which will continue this policy. The Committee is the first which, acting for the whole of London, "has aimed to give adequate relief to the needs of a worker who desires to keep his home together, and to leave him untroubled by the sense of obligation or loss of self-respect."

### Health Assurance.

DR. BOND, of Gloucester, has lately put forward the outline of a scheme which seems capable of useful development. He proposes that facilities should be afforded for enabling any persons so disposed to obtain trustworthy information as to the state of their health and as to their prospects of usefulness and longevity, and that this should be done by a mechanism somewhat resembling that of a life insurance society. It is suggested that the inquirer should fill up a form, framed somewhat on the general lines of a life insurance proposal, but with space for all the additional information bearing upon family history, past illnesses, and present state which it may be possible to obtain; and that the form, when completed, should be submitted to a medical practitioner for consideration and report. It is suggested that the two documents, taken together, the form and the report, would afford guidance of great value for many purposes—in relation to marriage, for example. They would, in point of fact, be important constituents of that life history of the individual which Mr. Galton has so often indicated as a thing eminently desirable of attainment; and, to secure their full usefulness, they should, of course, be written up from time to time until the last chapter has been reached. A great step would be made in the direction of "Eugenics," using the word in the modern or Galtonian sense of quality of offspring, as distinguished from the original sense in which it was applied to quantity only, if such reports could be compiled for every individual of a family, and preserved, together with permanent photographs and records of family events, in a volume which should at once fulfil the function of the "Family Bible" of our forefathers, and the more modern requirements arising from a recognition of the importance of a study of heredity. We hope that Dr. Bond may find it possible to bring his project into complete and workable form, and to render it generally available for the public.

### Promotion in the Army Medical Service.

THE nomination of Lieut.-Colonel Keogh, M.D., C.B., as Director-General of the Army Medical Service has excited the anger of those to whom promotion by seniority is a sacred principle. At the time we commented on the appointment, we hinted that it was probable an outcry might occur because the recommendation in the report of the War Office Reorganisation Committee that "no consideration except that of special fitness for the duties should arise" had been literally acted upon. We are not, therefore, surprised to be told in the columns of an influential contemporary that the choice is "very strongly condemned in professional quarters," the professional quarters including, of course, the disappointed aspirants to the office. The explicit complaint is, in fact, that a number of surgeons-general and lieutenant-colonels who are the seniors of the new director-general, were better qualified than he "by service and experience" for the command of the whole of the medical branch of the Army. This is the kind of objection which is always trotted out when any supposed vested interests are ignored for the benefit of the community. It is indisputable that Surgeon-General Keogh's period of service is considerably shorter, and that, in a sense, his experience has been less, than that of some of the officers who were passed over. But we rejoice that the so-called qualification of seniority was not allowed to prevail over all others. The importance of a particular age may be ridiculously exaggerated. Age-limits are purely artificial in character, and to bestow an appointment of supreme authority on a subordinate because of the length of his record of service is as unjustifiable as the silly rule which obtains in some quarters not to employ men over forty. As to the experience of the Director-General, the statement that he has "seen no war service," is singularly unfortunate in face of the honours conferred on him for the valuable work he did in the South African campaign, while it is obvious that he would not have been selected as deputy to Sir William Taylor if he had not manifested special capacity. Because we believe that his promotion is due neither to favouritism nor to social influence, is in defiance of red-tapeism, and may be attributed entirely to his own abilities, we signified approval when the announcement was made; and nothing that has been urged against it since has altered our opinion. We hope that it portends promotion by merit alone as the practice henceforth in the Army Medical Service.

